# Model-Predictive Control for Omnidirectional Mobile Robots in Logistic Environments Based on Object Detection Using CNNs

**DOI:** 10.3390/s23114992

**Published:** 2023-05-23

**Authors:** Stefan-Daniel Achirei, Razvan Mocanu, Alexandru-Tudor Popovici, Constantin-Catalin Dosoftei

**Affiliations:** 1 Department of Computer Engineering, “Gheorghe Asachi” Technical University of Iasi, 700050 Iasi, Romania; 2 Department of Automatic Control and Applied Informatics, “Gheorghe Asachi” Technical University of Iasi, 700050 Iasi, Romania

**Keywords:** omnidirectional mobile robots, object detection, convolutional neural networks, depth sensing, computer vision, discretized-time model, predictive control algorithm, navigation

## Abstract

Object detection is an essential component of autonomous mobile robotic systems, enabling robots to understand and interact with the environment. Object detection and recognition have made significant progress using convolutional neural networks (CNNs). Widely used in autonomous mobile robot applications, CNNs can quickly identify complicated image patterns, such as objects in a logistic environment. Integration of environment perception algorithms and motion control algorithms is a topic subjected to significant research. On the one hand, this paper presents an object detector to better understand the robot environment and the newly acquired dataset. The model was optimized to run on the mobile platform already on the robot. On the other hand, the paper introduces a model-based predictive controller to guide an omnidirectional robot to a particular position in a logistic environment based on an object map obtained from a custom-trained CNN detector and LIDAR data. Object detection contributes to a safe, optimal, and efficient path for the omnidirectional mobile robot. In a practical scenario, we deploy a custom-trained and optimized CNN model to detect specific objects in the warehouse environment. Then we evaluate, through simulation, a predictive control approach based on the detected objects using CNNs. Results are obtained in object detection using a custom-trained CNN with an in-house acquired data set on a mobile platform and in the optimal control for the omnidirectional mobile robot.

## 1. Introduction

The mobile robots sector has seen a global rise over the past decade. Industrial mobile robots are becoming more advanced to achieve higher levels of autonomy and efficiency in various industries [1]. These robots are equipped with sophisticated sensors, such as Light Detection and Ranging (LiDAR), stereo cameras, Inertial Measurement Unit (IMU), and a global positioning system or indoor positioning system, to gather information about the work environment and make well-informed decisions [2]. This is made possible by using complex algorithms for path planning, obstacle avoidance, and task execution. Furthermore, autonomous mobile robots, grouped in fleets, are often integrated with cloud-based technologies for remote monitoring and control, allowing for greater flexibility and scalability in their deployment.

Path planning is a crucial aspect of mobile robotics navigation because of the need to perform a task by moving from one point to another while avoiding obstacles and satisfying more constraints, among which are time, the level of autonomy given by the energy available, and significantly, maintaining safety margins regarding human operators and transported cargo. Mobile robot navigation is still one of the most researched topics of today, addressing two main categories: classical and heuristic navigation. In the variety of classical approaches, the most well-known algorithms, characterized by limited intelligence in [3], are cell decomposition, roadmap approach, and artificial potential field (APF). Heuristic approaches are more intelligent, including but not limited to the main components of computational intelligence (i.e., fuzzy logic, neural networks, and genetic algorithms). Researchers investigate solutions based on the particle swarm optimization algorithm, the FireFly algorithm, and the artificial BEE colony algorithm [4]. Combining classical and heuristic approaches, known as hybrid algorithms, offers better performances, especially for navigation in complex, dynamic environments [3].

In dynamic operational environments, the increased flexibility of autonomous mobile robots compared to automated guided vehicles is an added advantage due to decreased infrastructure setup and maintenance costs. Supplementary, the omnidirectional mobile robots (OMR), compared to other traction and steering systems (e.g., differential drive and Ackerman), provide three independent degrees of freedom (longitudinal and lateral translation, together with in-place rotation), motions that can be combined within certain speed and acceleration limits without producing any excessive wear on the ground contact surfaces. On the other hand, to obtain precise motion for the OMR, certain constraints apply to their suspension system and the smoothness of the ground surface.

Considering the computational requirements criteria, the planning technology of a mobile robot is divided into offline planning and online planning [5]. In offline planning, the path for the robot is pre-computed and stored in the robot’s memory. The robot then follows the pre-computed path to reach its destination. This approach is suitable for deterministic environments with a priori information. When the mobile robot navigates and performs tasks in a dynamic and uncertain environment, it is necessary to use the online planning approach. The robot computes its path in real time based on its current location and the information obtained from its perception module.

Independent of the type of path planning algorithm, the OMR structure is beneficial because it better resembles the material point model used for simplifying the modeling of robots in motion planning simulations. In [6,7,8,9], a four-wheel’s dynamic and kinematic modeling, OMR was studied using the Lagrange framework. Sliding mode control allows robust control for OMRs employing mecanum-wheels and rejects disturbances caused by unmodeled dynamics [10,11,12]. The nonholonomic model of the wheel was used to develop the dynamic equation of an OMR with four mecanum wheels [13]. The kinematic model of a three-wheeled mobile robot was used to create a predictive control model and filtered Smith predictor for steering the robot along predetermined paths [14]. A reduced dynamic model of the robot is the basis for developing a nonlinear model-predictive controller for trajectory tracking of a mecanum wheeled OMR [15]. A constrained quadratic programming problem is formulated towards optimizing the trajectory of a four-wheel omnidirectional robot [16]. Dynamic obstacles are considered in the work of the authors [17], whereas the numerical implementation presented in [18] is based on a three-wheeled omnidirectional robot. Distributed predictive control on a cooperative paradigm is discussed for a coalition of robots [19]. A nonlinear predictive control strategy with a self-rotating prediction horizon for OMR in uncertain environments is discussed. The appropriate prediction horizon was selected by incorporating the effects of moving velocity and road curvature on the system [20]. Adaptive model-predictive control, with friction compensation and incremental input constraints, is presented for an omnidirectional mobile robot [21]. Wrench equivalent optimality is used in a model-predictive control formulation to control a cable-driven robot [22]. Authors discuss an optimal controller to control the robot’s motion on a minimum energy trajectory [23]. Recently, potential field methods have been used mainly due to their naturally inspired logic. These methods are also widely used in omnidirectional mobile robots due to their simplicity and performance in obstacle avoidance [24,25]. Timed elastic-band approaches utilize a predictive control strategy to steer the robot in a dynamic environment to tackle real-time trajectory planning tasks [26]. Because the optimization is confined to local minima, the original timed elastic-band planner may cause a route through obstacles. Researchers proposed an improved strategy for producing alternate sub-optimal trajectory clusters based on unique topologies [27]. To overcome the mismatch problem between the optimization graph and grid-based map, the authors suggested an egocentric map representation for a timed elastic band in an unknown environment [28]. These path-planning methods are viable and pragmatic, and acquiring a desired path in various scenarios is generally possible. Yet, these approaches could have multiple drawbacks, such as a local minimum, a low convergence rate, a lack of robustness, substantial computation, and so on. Additionally, in logistic environments where OMR robots are equipped with conveyor belts to transport cargo, it is essential to guarantee low translational and rotational accelerations for the safety of the transported cargo. Therefore, we propose a nonlinear predictive control strategy on a reduced model where we can include maximum acceleration and velocities of the wheels within inequality constraints derived from the obstacle positions obtained from environment perception sensors (i.e., LiDAR and video camera). To tackle the problem of local minima, we propose a variable cost function based on the proximity of obstacles ahead to balance the global objectives.

### Object Detection for Mobile Platforms

Deep neural networks specifically created to analyze organized arrays of data (i.e., images) are known as convolutional neural networks, often called CNNs or ConvNets. CNNs offered solutions to computer vision challenges that are difficult to handle using conventional methods. They quickly advance to the state-of-the-art in areas such as semantic segmentation, object detection, and image classification. They are widely used in computer vision because they can quickly identify image patterns (such as lines, gradients, or more complex objects such as eyes and faces). CNNs are convolutional-layered feed-forward neural networks. CNNs attempt to mimic the structure of the human visual cortex with these specific layers.

Localization of object instances in images is implied by object detection. Object recognition generally assigns a class to the identified objects from a previously learned class list. Although object detection operates at the bounding-box level, it has no notion of different classes. The phrase “object detection” now encompasses both activities, even though they were initially two distinct jobs. So, before continuing, let’s be clear that object detection includes both object localization and object recognition.

Object detection and recognition is an essential field of study in the context of autonomous systems. The models can be broadly divided into one-stage and two-stage detectors. One-stage detectors are designed to detect objects in a single step, making them faster and more suitable for real-time applications, such as path planning based on object detection for a moving system. On the other hand, two-stage detectors use a two-step process, first proposing regions of interest and then looking for objects within those areas. This approach excludes irrelevant parts of the image, and the process is highly parallelizable. However, it comes at the cost of being slower than one-stage detectors.

To meet the constraints of the Nvidia Jetson mobile platforms considered for the OMR, lightweight neural networks were investigated for object detection and recognition. Among the models evaluated, YoloV5 [29], SSD-Mobilenet-v1 [30], SSD-Mobilenet-v2-lite [31] and SSD-VGG16 [32] were trained and tested. Earlier, the YoloV4 [33] model had already made significant improvements over the previous iteration by introducing a new backbone architecture and modifying the neck of the model, resulting in an improvement of mean average precision (mAP) by 10% and an increase in FPS by 12%. Additionally, the training process has been optimized for single GPU architectures, like the Nvidia Jetson family, commonly used in embedded and mobile systems.

A particular implementation is YoloV5 [29], which differs from other Yolo implementations using the PyTorch framework [34] rather than the original Yolo Darknet repository. This implementation offers a wide range of architectural complexity, with ten models available, starting from the YoloV5n (nano), which uses only 1.9M parameters, up to the YoloV5x6 (extra large), which uses 70 times as many parameters (140M). The lightest models are recommended for Nvidia Jetson platforms.

Recently, an increasing interest has been in developing mobile device object detection and recognition algorithms. A popular approach is using the Single Shot Detector (SSD) [35] neural network, a one-step algorithm. To further improve the efficiency of the SSD algorithm on mobile devices, researchers have proposed various modifications to the SSD architecture, such as combining it with other neural network architectures. One such modification is the use of the SSD-MobileNet and SSD-Inception architectures, which combine the SSD300 [35] neural network with various backbone architectures, such as MobileNet [30] or Inception [36]. These architectures, such as the Nvidia Jetson development platforms, are recognized for their real-time object detection capabilities on mobile devices.

These methods for object detection perform very well in general detection tasks. Yet, there must be more datasets and pretrained models for objects specific to the OMR environment, such as fixed or mobile conveyors, charging stations, other OMRs, etc. We have acquired our dataset and deployed domain-specific models for object detection in the OMR environment. We summarize the main contributions of this paper to the field of object detection and OMR control in logistic environments below:

- Acquisition of a data set for object detection in the OMR environment;
- Investigation of domain-specific models for object detection and providing a model to be used in an OMR environment;
- Deployed an image acquisition and object detection module fit for the real-time task of OMR control;
- Proposed a joint perception&control strategy based on a non-linear model-predictive control;
- Avoid local minima by using switched cost function weights to navigate around obstacles while still achieving the overall objective of decreasing travel distance;
- Guarantee maximum wheel speed and acceleration through the constrained non-linear MPC in order to ensure safe transportation of cargo;

The rest of the paper is organized as follows: Section 2 discusses object detection in the context of OMR’s logistic environment. First, some equipment experiments were conducted on the image acquisition sensor and the processing unit. We also describe the object detection dataset creation and object mapping in 2D and 3D perspectives. Section 3 is dedicated to the modeling and control of the OMR. We introduce the mathematical model used for developing the control strategy, followed by formulating the optimization problem considering the environmental objects. In the last two sections, we discuss the object detection results and the simulation of the control algorithm, conclude, and emphasize future work goals.

## 2. Object Detection for Omnidirectional Mobile Robots

### 2.1. Image Acquisition and Processing Unit

For the image acquisition unit, we analyzed four depth cameras. Depth information is needed to accurately place the detected objects on the 2D and 3D maps of the environment. The predictive control task relies on object maps. The most important features considered for the experiments were the correctness of the depth information and the integration of the camera with the Nvidia Jetson platforms, which are already in use on the Omnidirectional Robot.

**Figure 1:**
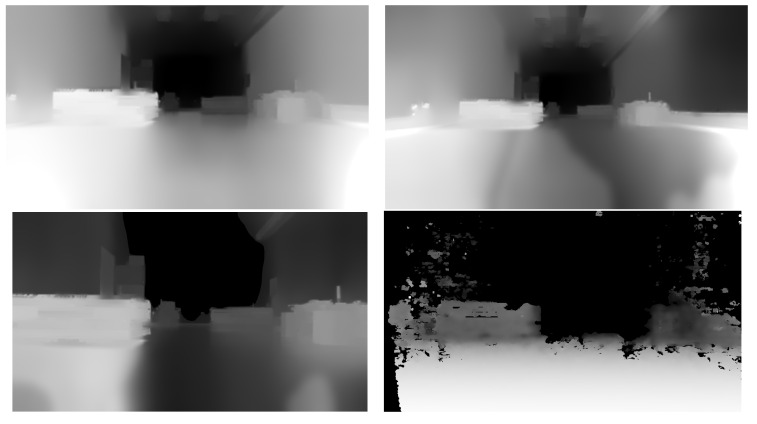
Depth information for ZED 1 (**top left**), ZED 2i (**top right**), ZED mini (**bottom left**), and Intel RealSense D435i (**bottom right**).

All Zed cameras perform well in indoor environments, but, as can be seen in Figure 1, the far-depth information provided by Zed 2i is significantly better. Depth information is completely missing after 10 m for Intel RealSense. The best depth information is given by Zed 2i; it also has the largest FoV. Based on the image acquisition experiments performed in the OMR environment, Zed 2i was chosen to be integrated into the robot.

Nvidia Jetson system-on-chip platforms are already used on the OMR. Some experiments evaluated the computational capabilities, detection precision, and the dependency between inference time and resolution. Localization is very important in our defined use cases for the OMR environment. MS COCO dataset [37] was used for object detection evaluation across different lightweight neural networks such as Mobilenet [30,31] and Yolo [29,33] which are suitable for mobile platforms.

The neural networks used for the first experiment are optimized using TensorRT to run on Jetson mobile platforms. In Table 1, we can see the run-time measurements for the selected models from the SSD family. The same solution takes considerably more time to run on the Jetson Nano.

A second experiment aims to see how the processing time evolves depending on the image resolution. Table 2 presents the results in terms of FPS on a test subset from Cityscapes data set [38,39]. The results emphasize that the inference time depends on the size of the images provided at input. Thus, the higher the image resolution, the slower the model. Jetson Xavier AGX is 4 to 6 times faster than Jetson Nano, depending on the model and the input resolution.

Following the analysis of hardware equipment and the experimental measurements, the Jetson architecture chosen to be integrated into the proposed solution for object detection in the OMR environment that meets the minimum requirements is the Nvidia Jetson Xavier AGX.

### 2.2. The Omnidirectional Robot Object Detection Dataset (OROD)

Enabling the efficient operation of autonomous robots is crucial for accurately detecting and recognizing objects specific to the OMR environment. Using the ZED 2i camera, we have acquired a new dataset for the object detection task that contains objects specific to the omnidirectional robot environment. The “Omnidirectional Robot Object Detection (OROD)” dataset includes charging stations, construction cones, mobile conveyors, and different types of fixed conveyors. The images in the dataset were captured using the camera mounted on an omnidirectional robot and were annotated with bounding boxes of objects. The dataset is intended to evaluate the performance of object detection algorithms in an omnidirectional robot environment.

**Figure 2:**
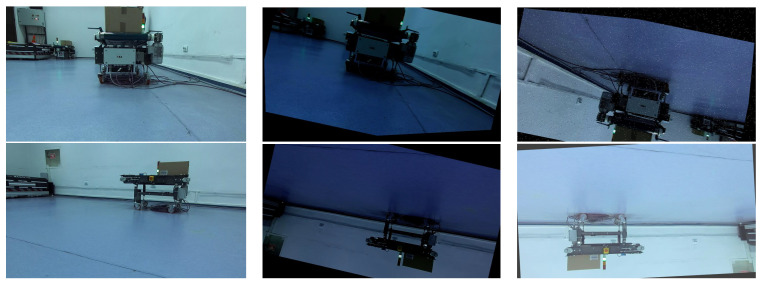
Acquired frame (**column 1**) and augmentation results (**columns 2 and 3**).

The OROD dataset contains 1343 images, each labeled with the objects of interest in the scene. The images were collected in different environments, such as industrial warehouses and logistics centers, to reflect the various scenarios in which an omnidirectional robot operates. Additionally, the data set includes images with varying lighting conditions, occlusions, and different orientations of the objects to represent real-world challenges in object detection. The training subset was augmented for better results by applying flip, rotation, zoom, hue, saturation, blur, noise, etc. The original and augmented data sets were split according to the figures from Table 3. Examples of the augmented images can be visualized in Figure 2. The dataset augmentation process did not change the initial class distribution; it scaled by 3.

The OROD dataset is the first to focus specifically on object detection in the context of an omnidirectional robot environment. It is intended to serve as a reference for evaluating the performance of object detection algorithms in this context and to promote research in this field.

The augmented data set and the raw dataset, both with YOLO annotations, are publicly available at https://universe.roboflow.com/gheorghe-asachi-technical-university-of-iasi/rmoa, accessed on 20 May 2023.

### 2.3. Detected Objects in 3D and Mapping

**Figure 3:**
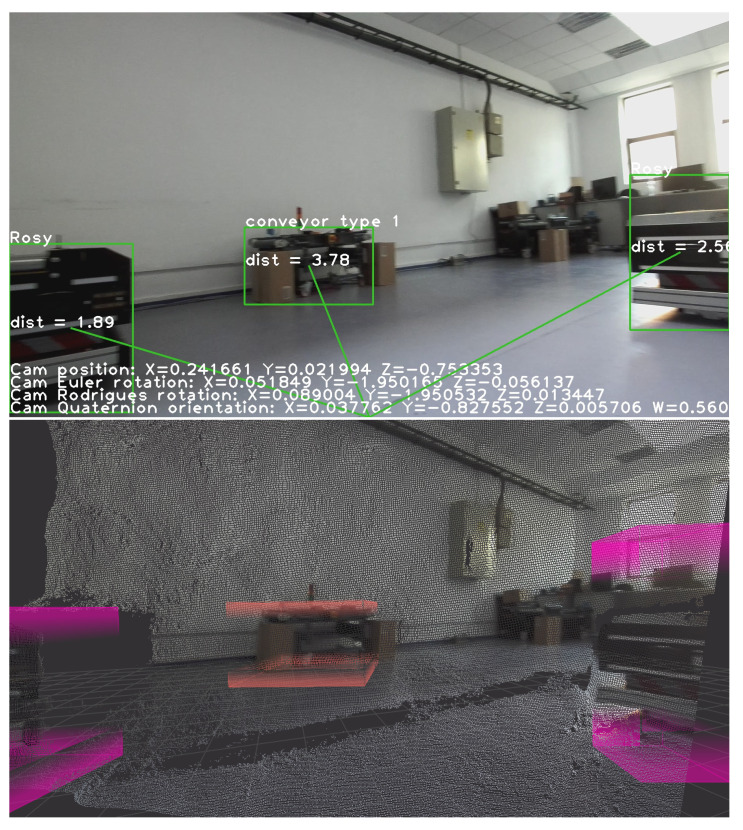
Object detection and distance estimation in meters (**top**) and 3D point cloud mapping (**bottom**).

All objects detected by the custom-trained model, along with the distances to them, are visible on the left side of Figure 3. Their 3D position is also exemplified on the right side. The distance between the scene object and the camera is measured from the back of the left eye of the camera and is given in meters.

In the context of an OMR moving through its environment, an important feature is to continuously be aware of its position and rotation relative to the starting point, the charging station, in our case.

**Figure 4:**
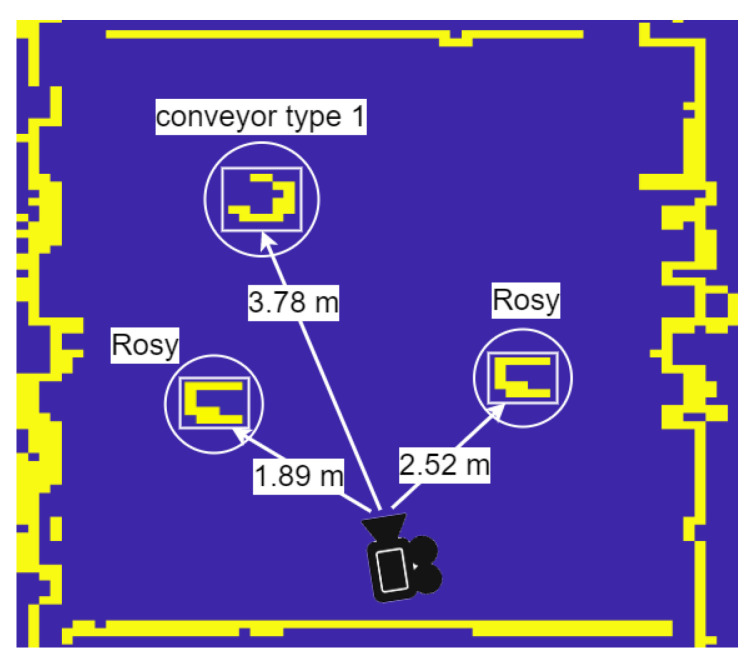
Bird’s eye view mapping of detected objects.

Examples of the OMR position and orientation are listed on the bottom of the frames in Figure 3. As a benefit of the IMU integration with Zed 2i, we can obtain the camera position, rotation, and quaternion orientation. In addition to the ZED 2i camera, the OMR is equipped with two LiDAR sensors for a 360-degree map. At this stage, the LiDAR data are empirically merged with the detected objects to obtain a bird’s-eye view map of the entire environment. In Figure 4, we can see the obtained map of the environment with the detected objects shown in Figure 3.

## 3. Model-Predictive Motion Control of OMR

We derive the motion control strategy of the OMR based on a non-linear optimization algorithm as the core of the motion controller. We define in Section 3.1 the mathematical model used in the predictions step of the controller. The continuous time equations are discretized by the Euler method to realize the numerical implementation. Then, we define the physical constraints of the robot’s actuators (i.e., omnidirectional wheel speed and acceleration) and the geometrical constraints of the objects (i.e., circumscribed circles of objects). We formulate the optimization problem considering the global objective of navigating on the shortest path, avoiding obstacles, and limiting the movement of the OMR within actuator limits.

### 3.1. Mathematical Model of 3D of Omnidirectional Robot

In this section, we define the discrete mathematical model used in the model-predictive controller to generate short-term paths and control the robot’s movement along the predicted trajectory. Equation (1) depicts the inverse kinematics matrix representation:

(1) $$\left[\begin{array}{c} \omega_{1}\\ \omega_{2}\\ \omega_{3}\\ \omega_{4} \end{array}\right]=J\left[\begin{array}{c} v_{x}\\ v_{y}\\ \Omega \end{array}\right]$$

where $v_{x}$ and $v_{y}$ are the longitudinal and lateral velocities of the OMR, respectively. Ω defines the angular speed along the normal axis, $\omega_{j},j=\bar{1..4}$ are the individual wheels’ angular velocities, while J is the inverse kinematic Jacobian matrix of the OMR defined in (2) [1]:

(2) $$J=\frac{1}{R}\left[\begin{array}{ccc} 1 & 1 & -(l_{x}+l_{y})\\ 1 & -1 & -(l_{x}+l_{y})\\ 1 & 1 & \left(l_{x}+l_{y}\right)\\ 1 & -1 & \left(l_{x}+l_{y}\right) \end{array}\right]$$

The forward kinematics of the 3DOF system are obtained from the lateral, longitudinal, and rotation velocities:

(3) $$\left[\begin{array}{c} \frac{dx}{dt}\\ \frac{dy}{dt}\\ \frac{d\theta }{dt} \end{array}\right]=\frac{R}{4}\left[\begin{array}{cccc} 1 & 1 & 1 & 1\\ 1 & -1 & 1 & -1\\ \frac{-1}{l_{x}+l_{y}} & \frac{-1}{l_{x}+l_{y}} & \frac{1}{l_{x}+l_{y}} & \frac{1}{l_{x}+l_{y}} \end{array}\right]\left[\begin{array}{c} \omega_{1}\\ \omega_{2}\\ \omega_{3}\\ \omega_{4} \end{array}\right]$$

where x,y,θ are plane coordinates and robot orientation, respectively. Moreover, *R* is the wheel radius, $l_{x}$ defines the distance from the GC to the front axle, while $l_{y}$ defines the half distance between the left and right wheels.

Pragmatically, it can be considered that deviations from the nominal kinematic model act on the system input. Therefore, we can design an input disturbance observer to compensate for unmodeled dynamics and disturbances. Let us define the disturbance acting on the system input as $F={\left[f_{1},f_{2},f_{3},f_{4}\right]}^{t}$, where the additive terms F act on the system inputs. The observer is designed considering the inverse kinematics of the process. An additional pole is added for the realizability of the observer. We define *Q* as a passive (i.e., unitary gain) first-order low-pass filter diagonal matrix. We define the estimated input disturbance as: $\hat{F}={\left[{\hat{f}}_{1},{\hat{f}}_{2},{\hat{f}}_{3},{\hat{f}}_{4}\right]}^{t}=-Q{\left[\omega_{1},\omega_{2},\omega_{3},\omega_{4}\right]}^{t}+QJ{\left[v_{x},v_{y},\dot{\theta }\right]}^{t}$ Therefore, the plant model becomes:

(4) $$\left[\begin{array}{c} \frac{dx}{dt}\\ \frac{dy}{dt}\\ \frac{d\theta }{dt} \end{array}\right]=\frac{R}{4}\left[\begin{array}{cccc} 1 & 1 & 1 & 1\\ 1 & -1 & 1 & -1\\ \frac{-1}{l_{x}+l_{y}} & \frac{-1}{l_{x}+l_{y}} & \frac{1}{l_{x}+l_{y}} & \frac{1}{l_{x}+l_{y}} \end{array}\right]\left[\begin{array}{c} \omega_{1}\\ \omega_{2}\\ \omega_{3}\\ \omega_{4} \end{array}\right]+\left[\begin{array}{c} f_{1}\\ f_{2}\\ f_{3}\\ f_{4} \end{array}\right]$$

The discretized-time model of (3) is obtained by backward rectangle area approximation (i.e., Euler method). Therefore, the system Equation (3) can be re-written in the state space framework $\dot{X}=AX+\left(J_{+}\right)\omega$, where the state transition matrix is null, the state vector is $X={\left[x\;\;\;y\;\;\;\theta \right]}^{t}$ while the input matrix $J_{+}$ is defined as $J_{+}={\left(J^{T}J\right)}^{-1}J^{T}$. Thus, we obtain the discretized-time model of the OMR in global coordinates:

(5) $$X_{k+1}=I_{3}X_{k}+\left(J_{+}\right)T_{s}\omega_{k}$$

where $I_{3}\in R^{3\times 3}$ unity matrix, $X_{k+1}={\left[x_{k}\;\;y_{k}\;\;\theta_{k}\right]}^{t}$ is the state vector at iteration k+1, $T_{s}$ is the sampling time and $\omega_{k}={\left[\omega_{1_{k}}\;\;\omega_{2_{k}}\;\;\omega_{3_{k}}\;\;\omega_{4_{k}}\right]}^{t}$ is the input vector.

(6) $$\left[\begin{array}{c} x_{k+1}\\ y_{k+1}\\ \theta_{k+1} \end{array}\right]=\left[\begin{array}{c} x_{k}\\ y_{k}\\ \theta_{k} \end{array}\right]+T_{s}\frac{R}{4}\left[\begin{array}{cccc} 1 & 1 & 1 & 1\\ 1 & -1 & 1 & -1\\ \frac{-1}{l_{x}+l_{y}} & \frac{-1}{l_{x}+l_{y}} & \frac{1}{l_{x}+l_{y}} & \frac{1}{l_{x}+l_{y}} \end{array}\right]\left[\begin{array}{c} \omega_{1_{k}}\\ \omega_{2_{k}}\\ \omega_{3_{k}}\\ \omega_{4_{k}} \end{array}\right]$$

To improve controller behavior w.r.t to deviations of the model and input perturbation, the extended discretized model can be used for states and output predictions within the MPC solver:

(7) $$\left[\begin{array}{c} x_{k+1}\\ y_{k+1}\\ \theta_{k+1} \end{array}\right]=\left[\begin{array}{c} x_{k}\\ y_{k}\\ \theta_{k} \end{array}\right]+T_{s}\frac{R}{4}\left[\begin{array}{cccc} 1 & 1 & 1 & 1\\ 1 & -1 & 1 & -1\\ \frac{-1}{l_{x}+l_{y}} & \frac{-1}{l_{x}+l_{y}} & \frac{1}{l_{x}+l_{y}} & \frac{1}{l_{x}+l_{y}} \end{array}\right]\left[\begin{array}{c} \omega_{1_{k}}\\ \omega_{2_{k}}\\ \omega_{3_{k}}\\ \omega_{4_{k}} \end{array}\right]+T_{s}\left[\begin{array}{c} f_{1_{k}}\\ f_{2_{k}}\\ f_{3_{k}}\\ f_{4_{k}} \end{array}\right]$$

Table 4 contains the parameters of the mobile robot and the sampling time considered for the time-discretization of the process.

### 3.2. OMR Motion Optimization Problem

In the optimization problem, we aim to find the solution at time $kT_{s}$, comprised of actuator commands $\omega_{j}^{\left(i,k\right)}\;\;for\;\;\;\;j=\bar{1..4},$ i∈{1…H} satisfying actuator physical constraints, the geometric constraints, and to fulfill the global objective of traveling the shortest distance and avoiding the detected obstacles. Therefore, we formulate the optimization problem as follows. Find,

(8) $$\begin{array}{cc} \min_{x\left(\cdot |k\right),\;\;y\left(\cdot |k\right),\;\;\theta \left(\cdot |k\right),\;\;d_{p}\left(\cdot |k\right)}\; & J_{k}\left(X,X_{r},\alpha \right)\\ s.t.-\omega_{UB} & \leq \omega_{j,j=\bar{1..4}}\leq \omega_{UB}\\ -a_{UB} & \leq {\dot{\omega }}_{j,j=\bar{1..4}}\leq a_{UB}\\ C_{o} & <0 \end{array}$$

where $J_{k}$ is the cost function defined in (9), x(·|k),y(·|k),θ(·|k) are the solutions of the optimization problem; $\omega_{UB}$ and $a_{UB}$ are the upper bounds of the angular velocity and acceleration of the wheels, respectively. $C_{o}$ is the geometric constraints vector and is defined in (22).

The cost function $J_{k}$ is defined by:

(9) $$\begin{array}{c} J_{k}\left(X,X_{r},\alpha \right)=\frac{1}{2}\sum_{i=0}^{H-1}[w_{x}\left(\alpha \right){\left(x_{r}^{\left(i|k\right)}-x^{\left(i|k\right)}\right)}^{2}+w_{y}\left(\alpha \right){\left(y_{r}^{\left(i|k\right)}-y^{\left(i|k\right)}\right)}^{2}+w_{\theta }{\left(\theta_{r}^{\left(i|k\right)}-\theta^{\left(i|k\right)}\right)}^{2}+ \end{array}$$

(10) $$\begin{array}{c} +w_{Tx}{\left(x_{r}^{\left(H-1|k\right)}-x^{\left(i|k\right)}\right)}^{2}+w_{Ty}{\left(y_{r}^{\left(H-1|k\right)}-y^{\left(i|k\right)}\right)}^{2}]+w_{p}\left(\alpha \right)\sum_{i=0}^{H-1}\left(d_{p}{\left(X,X_{0},X_{f}\right)}^{\left(i|k\right)}\right) \end{array}$$

where $X_{r}\in R^{H\times 3}$ is the reference trajectory matrix of the OMR over the prediction horizon *H*:

(11) $$X_{r}=\left[\begin{array}{ccc} x_{r}^{\left(0,k\right)} & y_{r}^{\left(0,k\right)} & \theta_{r}^{\left(0,k\right)}\\ x_{r}^{\left(1,k\right)} & y_{r}^{\left(1,k\right)} & \theta_{r}^{\left(1,k\right)}\\ ... & ... & ...\\ x_{r}^{\left(H-1,k\right)} & y_{r}^{\left(H-1,k\right)} & \theta_{r}^{\left(H-1,k\right)} \end{array}\right]$$

$d_{p}\left(X_{0},X_{f}\right)$ is the length of the projection of the OMR geometric center over the ideal straight path connecting the starting (i.e., initial) node with the final node and is defined in (12):

(12) $$d_{p}\left(X,X_{0},X_{f}\right)=\sqrt{|L_{1}^{2}-{\left[\left(L_{1}^{2}+L_{3}^{2}-L_{2}^{2}\right)/\left(2L_{3}\right)\right]}^{2}|}$$

with

(13) $$\begin{array}{ccc} L_{1} & = & \sqrt{{\left(x-x_{0}\right)}^{2}+{\left(y-y_{0}\right)}^{2}} \end{array}$$

(14) $$\begin{array}{ccc} L_{2} & = & \sqrt{{\left(x-x_{r}^{\left(H-1\right)}\right)}^{2}+{\left(y-y_{r}^{\left(H-1\right)}\right)}^{2}} \end{array}$$

(15) $$\begin{array}{ccc} L_{3} & = & \sqrt{{\left(x_{0}-x_{r}^{\left(H-1\right)}\right)}^{2}+{\left(y_{0}-y_{r}^{\left(H-1\right)}\right)}^{2}} \end{array}$$

where $L_{1}$, $L_{2}$, and $L_{3}$ define the L2-norms between the OMR position, initial, and resting positions, while $X_{0}={\left[x_{0},y_{0},\theta_{0}\right]}^{t}$ and $X_{f}={\left[x_{r}^{\left(H-1\right)},y_{r}^{\left(H-1\right)},\theta_{r}^{\left(H-1\right)}\right]}^{t}={\left[x_{f},y_{f},\theta_{f}\right]}^{t}$ are the initial and final resting positions. In the cost function, we aim to penalize by weights $w_{x}\left(\alpha \right)$ and $w_{y}\left(\alpha \right)$ the deviation from the reference trajectory $x_{r}^{\left(i\right)},y_{r}^{\left(i\right)},i=\bar{0..H-1}$ defined by (25); by weight $w_{\theta }$ it is penalized the deviation from the desired orientation of the OMR. The set-point orientation $\theta_{r}^{\left(i\right)},i=\bar{0..H-1}$ is such that the OMR remains with the frontal part facing the destination location. By $w_{Tx}$, we penalize the terminal cost of $x_{r}$ and $y_{r}$ to reduce the steady-state error. Therefore, $w_{Tx}>w_{x}$ and $w_{Ty}>w_{y}$; $w_{p}\left(\alpha \right)$ is a weight with two discrete states, and its value is a function of α which depends on the proximity (tolerance) of the closest object and is defined by (23).

The actuator constraints of the OMR are defined as:

(16) $$\begin{array}{cc} -\omega_{UB} & \leq \omega_{j,j=\bar{1..4}}\leq \omega_{UB} \end{array}$$

(17) $$\begin{array}{cc} -a_{UB} & \leq {\dot{\omega }}_{j,j=\bar{1..4}}\leq a_{UB} \end{array}$$

We define the physical-space constraints from the coordinates of the objects and their known sizes as:

(18) $$\begin{array}{c} -{\left(x-x_{o}\right)}^{2}-{\left(y-y_{o}\right)}^{2}+r_{o}^{2}\leq 0 \end{array}$$

where $x_{o}={\left[x_{o_{1}}\cdots x_{o_{no}}\right]}^{t}$ and $y_{o}={\left[y_{o_{1}}\cdots y_{o_{no}}\right]}^{t}$ are the coordinates $r_{o}={\left[r_{o_{1}}\cdots r_{o_{no}}\right]}^{t}$ is the radius of the circle circumscribed about the polygon defining the object. Index no refers to *Number-of-Objects*, while, index o signifies the word Objects, also, index *a* refers to word *Actuators*. The global coordinates of the obstacles are obtained from the local coordinates of the OMR according to the equation below:

(19) $$\left[\begin{array}{c} x_{o}\\ y_{o} \end{array}\right]=\left[\begin{array}{cc} cos\phi & sin\phi \\ -sin\phi & cos\phi \end{array}\right]\left[\begin{array}{c} \left[\begin{array}{c} x_{l}\\ y_{l} \end{array}\right]-\left[\begin{array}{c} x\\ y \end{array}\right] \end{array}\right]$$

where ϕ is the angle from the global system’s abscissa to the local system’s abscissa. $x_{l}$ and $y_{l}$ are the local coordinates of the detected objects, and x and y are the global coordinates of the local system’s origin. From inequality constraints (16) and (18), we obtain a concatenated vector of inequality constraints denoted by $C_{k}\in R^{(n_{a}\cdot n_{w}\cdot H+n_{o}\cdot H)\times 1}\leq 0$:

(20) $$C_{k}={\left[C_{a}^{t},C_{o}^{t}\right]}^{t}\in R^{(n_{a}\cdot n_{w}\cdot H+n_{o}\cdot H)\times 1}$$

where $C_{a}\in R^{n_{a}\cdot n_{w}\cdot H\times 1}$, $C_{o}\in R^{n_{o}H\times 1}$ are defined below:

(21) $$C_{a}^{\left(k\right)}=\left[\begin{array}{c} |\omega_{1}^{\left(0|k\right)}|-\omega_{UB}\\ |\omega_{1}^{\left(1|k\right)}|-\omega_{UB}\\ \vdots \\ |\omega_{1}^{\left(H-1|k\right)}|-\omega_{UB}\\ |\omega_{2}^{\left(0|k\right)}|-\omega_{UB}\\ \vdots \\ |\omega_{4}^{\left(H-1|k\right)}|-\omega_{UB}\\ |{\dot{\omega }}_{1}^{\left(0|k\right)}|-a_{UB}\\ \vdots \\ |{\dot{\omega }}_{4}^{\left(H-1|k\right)}|-a_{UB} \end{array}\right]\leq 0$$

(22) $$C_{o}^{\left(k\right)}=\left[\begin{array}{c} -{\left(x^{\left(0|k\right)}-x_{o_{1}}\right)}^{2}-{\left(y^{\left(0|k\right)}-y_{o_{1}}\right)}^{2}+r_{o_{1}}^{2}\\ -{\left(x^{\left(1|k\right)}-x_{o_{1}}\right)}^{2}-{\left(y^{\left(1|k\right)}-y_{o_{1}}\right)}^{2}+r_{o_{1}}^{2}\\ \vdots \\ -{\left(x^{\left(H-1|k\right)}-x_{o_{1}}\right)}^{2}-{\left(y^{\left(H-1|k\right)}-y_{o_{1}}\right)}^{2}+r_{o_{1}}^{2}\\ -{\left(x^{\left(0|k\right)}-x_{o_{2}}\right)}^{2}-{\left(y^{\left(0|k\right)}-y_{o_{2}}\right)}^{2}+r_{o_{2}}^{2}\\ \vdots \\ -{\left(x^{\left(H-1|k\right)}-x_{o_{no}}\right)}^{2}-{\left(y^{\left(H-1|k\right)}-y_{o_{n_{o}}}\right)}^{2}+r_{o_{no}}^{2} \end{array}\right]\leq 0$$

In the previous equations, $n_{a}=2$ defines the number of constraints regarding actuators, and it is two because we included two types of actuator restrictions: angular speed and angular acceleration.

We approximate numerically $\dot{\omega_{j}}$ by $\dot{\omega_{j}}\approx \frac{\left(\omega_{j}^{\left(i\right)}-\omega_{j}^{\left(i-1\right)}\right)}{T_{s}}$ where $T_{s}$ is the sampling time.

In the cost function (9), we propose that $w_{p}\left(\alpha \right)$, $w_{x}\left(\alpha \right)$ and $w_{y}\left(\alpha \right)$ are switched between their two states based on the value of $\alpha =max_{1\leq j\leq n_{o}\cdot H}\;C_{o_{j}}$ which, practically, determines the minimum proximity to an obstacle from the object list. Therefore,

(23) $$\begin{array}{c} w_{p}\left(\alpha \right)=\left\{\begin{array}{cc} w_{p_{1}}, & \mathrm{if}\;\alpha >tol\\ w_{p_{2}}, & \mathrm{if}\;\alpha \leq tol \end{array}\right.\\ w_{x}\left(\alpha \right)=w_{y}\left(\alpha \right)=\left\{\begin{array}{cc} w_{{xy}_{1}}, & \mathrm{if}\;\alpha >tol\\ w_{{xy}_{2}}, & \mathrm{if}\;\alpha \leq tol \end{array}\right. \end{array}$$

In the previous equation, tol defines the avoidance tolerance.

The set-point orientation over the control horizon *H* is defined as:

(24) $$\theta_{r}^{\left(i\right)}=atan_{2}\left(y_{r}^{\left(H-1\right)}-y,x_{r}^{\left(H-1\right)}-x\right)\frac{360}{2\pi },\mathrm{for}\;i=\bar{0..H-1}$$

and the reference trajectory is given by a first-order static function where the slope λ and the bias ρ are given by:

$$\lambda =\left\{\begin{array}{cc} \frac{y_{f}-y}{x_{f}-x} & ,\;\mathrm{if}\;x_{f}\neq x\\ 0, & \mathrm{otherwise} \end{array}\right.$$

$$\rho =\left\{\begin{array}{cc} y_{f}-\frac{y_{f}-y}{x_{f}-x}x_{f} & ,\;\mathrm{if}\;x_{f}\neq x\\ 0, & \mathrm{otherwise} \end{array}\right.$$

(25) $$\begin{array}{ccc} x_{r}^{\left(i\right)} & = & \frac{x_{f}-x}{H}i+x\;\;,i=\bar{0..H-2}\\ x_{r}^{\left(H-1\right)} & = & x_{f}\\ y_{r}^{\left(i\right)} & = & \lambda x_{f}^{\left(i\right)}+\rho \;\;,i=\bar{0..H-2}\\ y_{r}^{\left(H-1\right)} & = & y_{f} \end{array}$$

### 3.3. Control Algorithm—One Step Optimization

**Figure 5:**
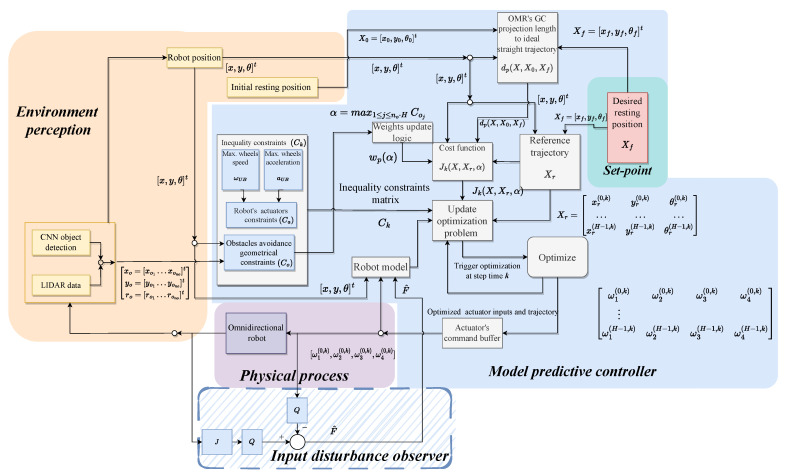
Illustrative block diagram of the control structure.

The control algorithm core is the sequential quadratic optimizer with a constraint tolerance of $1.0\times 10^{-3}$ and an optimality tolerance of $1.0\times 10^{-4}$ deduced heuristically through multiple experiments. Under this parametrization, the behavior is fairly robust and predictable with respect to the initial robot position, final resting position, varying size obstacles, wheel speeds, and acceleration. The object lists consist of a matrix of object positions obtained from the perception module. In order to determine the radius of the obstacles, we use the Moor–Neighbour tracing algorithm with Jacob’s stopping criteria, which provides the contour of the objects from LIDAR data. Beyond providing LIDAR data, CNN can provide estimates of object radius with higher precision based on the object class. In order to reduce the computation time, the optimization problem is reformulated at each sampling time, and we consider in the optimization only those objects within a maximum radius ($d_{max}$) relative to the OMR’s geometric center. The avoidance radius for each object is determined from the actual object radius with an additional tolerance according to OMR’s dimensions. The reference orientation $\theta_{r}$, and reference trajectory $\left(x_{r},y_{r}\right)$ are determined at each sample time since the OMR position evolves from one pose to another, constantly changing the heading to the final resting position. The first computed command over the predicted horizon is applied to the process inputs. We summarize the control algorithm steps for one sampling time $T_{s}$ in Algorithm 1.
**Algorithm 1** Main control algorithm**Inputs:***Desired setpoint $X_{f}$ from mission planner*, $X_{f}\leftarrow {\left[x_{f}\;\;\;y_{f}\;\;\;\theta_{f}\right]}^{t}$;*Initial position $X_{0}$ from perception module*, $X_{0}\leftarrow {\left[x_{0}\;\;\;y_{0}\;\;\;\theta_{0}\right]}^{t}$;**Outputs:***Actuator commands over horizon H, $\omega_{j}^{\left(i,k\right)},j=\bar{1..4},i=\bar{0..H-1}$**Predicted path over horizon H, $x^{\left(i|k\right)},y^{\left(i|k\right)},\theta^{\left(i|k\right)},i=\bar{0..H-1}$****Runtime****Acquire object list data: positions ($x_{o},y_{o}$), radius ($r_{o}$) from perception module*;*Detect object boundaries from LiDAR data using Moore-Neighbor tracing algorithm with Jacob’s stopping criteria [40]**[B,L] = bwboundaries(LiDAR data); (Matlab specific function)*l = 0;**for** k∈{1…length(B)} **do**    Objectboundary←B{k}; *B is Matlab cell data-type, therefore brackets are ’{}’ for indexing*    *Ignore objects composed of a very small or very high number of pixels (usually are artifacts or room boundaries)*    **if** $Boundary_{Min}\leq numel\left(Object\;boundary\right)/2\leq Boundary_{Max}$ **then**        l←l+1;        *If the number of objects exceeds buffer size (MaxNoObjs), an error will be thrown, and optimization will not be started*        **if** l>MaxNoObjs **then**           l←−1;           break;        $\bar{xy}\leftarrow mean\left(Object\;boundary\right)\in R^{2\times 1}$ *Matlab specific function to determine mean value over each line of a matrix.*        $x_{o}\left(l\right)\leftarrow \bar{xy}\left[2\right];$        $y_{o}\left(l\right)\leftarrow \bar{xy}\left[1\right];$        $r_{o}\left(l\right)\leftarrow max(|max\left(Objectboundary\right)-min\left(Objectboundary\right)|);$*Matlab specific functions to determine min, max values of matrix rows; or $r_{o}$ provided by CNN subsystem;*noObjs←l; *No. of all objects detected in the map*;*Determine relevant objects (within specified proximity $d_{max}$)*;**for** k∈{1…noObjs} *No. of all objects* **do**    *Calculate distance to each relevant object:*    $d_{o}\leftarrow \sqrt{{\left(x_{o}\left[k\right]-x\right)}^{2}+{\left(y_{o}\left[k\right]-y\right)}^{2}}$;    **if** $d_{o}\leq d_{max}$ **then**        *Update object radius to include tolerance w.r.t to OMR dimensions*        $r_{o}\left[k\right]\leftarrow r_{o}\left[k\right]+max\left(l_{x},l_{y}\right)$;        $n_{o}\leftarrow n_{o}+1;$*Calculate reference trajectory $x_{r}^{\left(i\right)},y_{r}^{\left(i\right)}$ according to Equation (25)*;*Calculate reference angle $\theta_{r}^{\left(i\right)},i=\bar{0..H-1}$ according to Equation (24)*;*Input data to optimizer: Sampling time: $T_{s}$; Object list: $x_{o},y_{o},r_{o}$; Number of objects $n_{o}$, Initial resting point $X_{0}$; Final resting point $X_{f}$, Run-time reference trajectory $X_{r}$; Previous optimized commands $\omega_{i}$**Optimize;**Save in buffer the optimized commands;**Provide to the process inputs the first (i.e., i = 0) command from the- control buffer*;

Figure 5 depicts the control structure consisting of two main subsystems: Environment perception, Model-Predictive Controller, and the interconnection with the psychical process.

**Figure 6:**
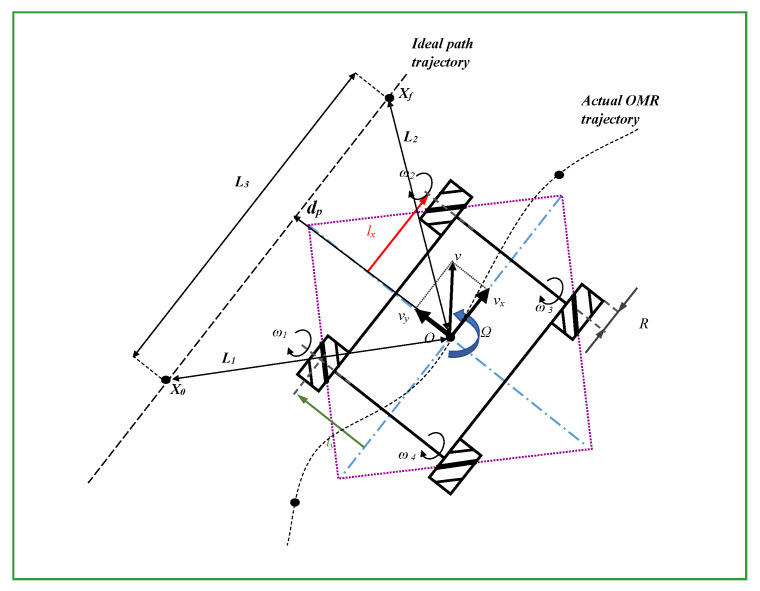
Coordinates system for control algorithm illustrating the used notations.

Figure 6 illustrates the main coordinates and notations used throughout the optimization problem. The projection $d_{p}$ from the robot CG to the imaginary straight path connecting the initial $X_{0}$ and final $X_{f}$ resting locations is noticeable. Moreover, the L2-norms used in calculating the cost function, $L_{1}$, $L_{2}$, and $L_{3}$ define the distances between the OMR, initial, and resting positions.

Table 5 contains the parameters of the model-predictive controller, including the penalizing factor of the cost function, the proximity threshold (*tol*) for switching cost function weights, the radius w.r.t to OMR’s CG to and the prediction horizon.

## 4. Results

### 4.1. Object Detection Results

The performance of the selected object detection solutions (ssd-mobilenet-v1, ssd-mobilenet-v2-lite, ssd-vgg16, and YoloV5) was evaluated on a testing subset with image resolutions varying between 720 × 404 and 2048 × 1024 pixels. The neural networks were tested on the Nvidia Jetson AGX mobile platform with the same input.

All models are optimized for Jetson Xavier AGX with the TensorRT framework from CUDA for Nvidia cards. The run time of the three selected architectures from the SSD family and the five main YoloV5 [29] is presented in Table 6. Architectures with fewer parameters performed better in terms of frames per second. Being the lightest model, the Nano YoloV5 is six times faster than the Extra Large model, the largest we considered for the Jetson platform. This highlights the importance of considering the specific hardware platform and the model’s complexity for deploying object detection algorithms on mobile robots.

The two largest YoloV5 models did not bring any improvements for the overall precision and the precision per class compared to the Medium architecture; therefore, they were not considered for Table 6. A comparison between the precision of the models can be made based on the figures presented in Table 7. All architectures were trained for 150 epochs to evaluate the mean Average Precision. SSD Mobilenet v2 lite and SSD VGG16 reach a similar mAP@0.5 of 98–99%, while SSD Mobilenet v1 has a lower precision on the test subset, 86%.

**Figure 7:**
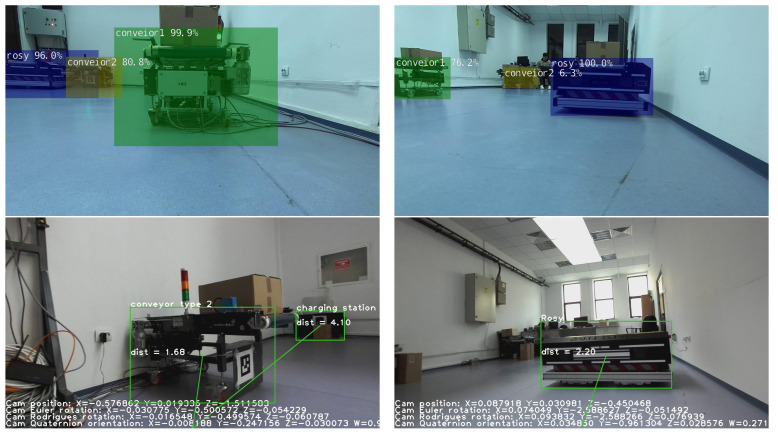
Detected objects with SSD architectures (**1st row**) and with YOLOv5 architecture (**2nd row**).

Based on the results from Table 6 and Table 7, we can draw the conclusion that the best model for our OMR object detection use cases is YoloV5 Medium which has a mAP comparable to SSD-VGG16, with the benefit of being twice as fast. Detection examples with the neural network models tested in the OMR environment are shown in Figure 7.

### 4.2. Simulation Results

To evaluate the control performances, we considered scenarios where the initial and final positions varied throughout the room so that obstacles blocked the OMR path. We perform numerical simulations on real data acquired from the perception module. We evaluate the steady-state error, the possible constraint violations, the cost function, and the optimization run-time.

**Figure 8:**
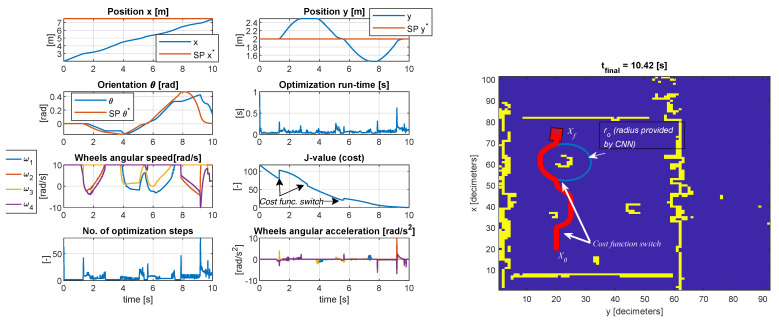
Simulation results of the model−predictive controller with LiDAR data and simulation of camera detection (test case I).

In the first test case considered in Figure 8, the final resting position $X_{f}$ is reached after avoiding the two obstacles on the circumference of virtual circles centered around the objects. The inequality geometric inequality constrained $C_{o}<0$, and the actuator constraints are satisfied $C_{a}<0$ with an acceptable tolerance. Generally, the tolerance is within the expected margin of $1.0\times 10^{-3}$. The steady-state error of the controlled position (x,y) is less than 1% as measured around moment t=10.2 s. The transient time is limited by the upper and lower bounds of the wheel speed, in this case, ±10 rad/s. The orientation θ changes at each sample time as the vehicle travels towards $X_{f}$. Hence, the tracking is decent, with a peak error of 17 degrees noticeably on the roundabouts of the objects since the optimizer is more constrained. The cost function decreases as the vehicle evolves across the map. In the proximity of an object, the cost function is purposely increased to avoid local minima by amplifying the deviation from the reference trajectory and decreasing the penalizing weight for the projection to the ideal path to allow solutions on the circumference of the encircled object. The maximum number of iterations was 79 with a run-time of 0.8945 s, and the minimum number of iterations was 2 with a run-time of 0.0204 s (CPU Intel i7 7500u, dual-core, 7th generation). The mean number of iterations was 6.526, with an execution time of 0.0647 s. It must be mentioned that the run-time is less relevant since in MEX mode (Matlab executable), the run-time can be reduced considerably (in MEX mode, the average run-time was 0.0507 s, while in normal mode 0.0647 s). The execution time is platform dependent.

**Figure 9:**
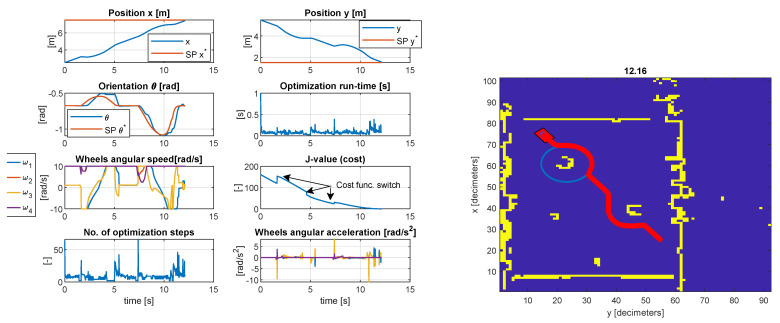
Simulation results of the model−predictive controller with LiDAR data and simulation of camera detection (test case II).

In the second scenario presented in Figure 9, the behavior is similar concerning the constraint tolerances. The violation of the object boundaries is within the expected limit, and the steady-state error of the controlled pose (x,y,θ) is less than 1%. In this case, the actuator constraints limit the transient time, ±10 rad/s. The maximum number of iterations was 66 with a run-time of 0.9923 s, and the minimum number of iterations was 2 with a run-time of 0.022 s (same CPU as mentioned in test case I). The mean number of iterations was 8.5658, with a mean execution time of 0.0814 s. In MEX mode, the maximum run-time was 0.5681 s, the minimum 0.0039 s, and the average 0.0356 s. Generally, the behavior is as expected, and the run-time proves the applicability of the control structure.

**Figure 10:**
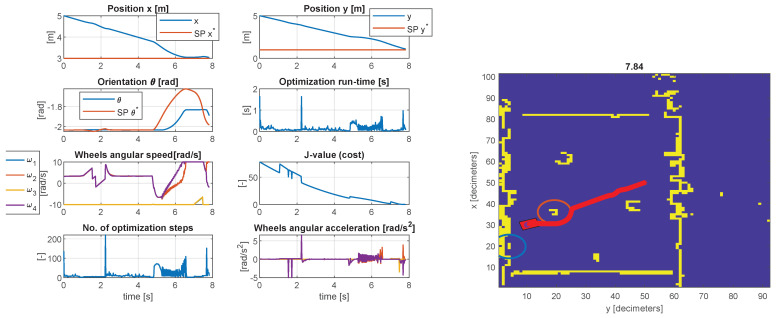
Simulation results of the model−predictive controller with LiDAR data and simulation of camera detection (test case III).

Similar behavior is obtained in the third test case presented in Figure 10, but the maximum run-time is slightly higher at 1.6 s, the maximum number of iterations is 210, and the minimum is 2. The minimum run-time was 0.0191 s. However, the average run-time in MEX mode is 0.0358 s with a maximum of 0.3176 s (instead of 1.6 s as in normal mode) and a minimum of 0.0043 s.

## 5. Conclusions and Future Work

The use of CNNs for object detection in mobile robot navigation provides benefits such as accuracy, robustness, and adaptability, which are desirable for the navigation of mobile robots in a logistic environment.

The paper proves the use of an object detector for a better understanding of the OMR working environment. To overcome this challenge, we also acquired a dataset for domain-specific object detection that was made public. It contains all objects of interest for the working environment, such as fixed or mobile conveyors, charging stations, other robots, and boundary cones. The results show a detection accuracy of 99% using the selected lightweight model, which was optimized to run on the available mobile platform already installed on the OMR at about 109 frames per second. The detection results offer a better understanding of the LiDAR map by assigning a name to obstacles and objects within the working environment, allowing the control model constraints to be adjusted on the fly.

This paper also demonstrates the model-predictive control of the OMR in logistic environments with actuator and geometric constraints. We avoid local minima by using variable cost function weights to navigate around obstacles while still achieving the overall objective of reducing travel distance. The execution runtime of the optimizer allows for practical implementation while the control performance is within the expected margin.

Future work is also expected to involve the deployment of the OMR controller and testing in a controlled environment and then in an automated logistic warehouse. One of the short-term goals is to collect and annotate more instances of domain-specific objects so that the intraclass variety is better covered and the detector can extrapolate on new data.

## Figures and Tables

**Table 1:** Object detection evaluation of the SSD model family.

Architecture	FPS on Jetson	FPS on Jetson
	Nano	Xavier AGX
SSD–Mobilenet–v1	10	83
SSD–Mobilenet–v2	7	61
SSD–Inception–v2	6	42

**Table 2:** Inference Time vs. Image Resolution.

Architecture	Resolution	FPS on Jetson	FPS on Jetson
		Nano	Xavier AGX
SSD–Mobilenet–v1	2048 × 1024	15 fps	91 fps
	1024 × 512	23 fps	102 fps
SSD–Mobilenet–v2	2048 × 1024	12 fps	74 fps
	1024 × 512	18 fps	76 fps
SSD–Inception–v2	2048 × 1024	11 fps	63 fps
	1024 × 512	15 fps	63 fps

**Table 3:** OROD train-val-test split.

	Annotated Frames before/after Augmentation	Percentage before/after Augmentation
train-initial	940/2816	70/88
validation	269/269	20/8
test	134/134	10/4

**Table 4:** OMR parameters.

Parameter	Value
Wheel radius (*R*)	0.076 [m]
Distance from GC to front axle ($l_{x}$)	0.294 [m]
Half distance between left and right wheels ($l_{y}$)	0.2 [m]
Sampling time ($T_{s}$)	0.02 [s]

**Table 5:** Control parameters.

Parameter	Value
Cost weight 1 of reference trajectory ($w_{xy1}$)	0.6
Cost weight 1 of projection length to ideal path ($w_{p_{1}}$)	0.01
Cost weight 2 of reference trajectory ($w_{xy2}$)	0.05
Cost weight 2 of projection length to ideal path ($w_{p_{2}}$)	2.0
Cost weight of orientation angle ($w_{\theta }$)	0.3
Terminal cost weight of reference trajectory ($w_{Tx}$)	0.8
Terminal cost weight of reference trajectory ($w_{Ty}$)	0.8
Threshold for switching cost weights *tol*	−0.1 [m]
Maximum distance from CG to objects ($d_{max}$)	2.5 [m]
Prediction horizon (*H*)	10 [samples] ($T_{horizon}$ = 0.2 s)
Map cell size	10 [cm]

**Table 6:** Inference time.

Architecture	FPS on Jetson Xavier AGX	Number of Parameters
SSD Mobilenet v1	120	4.2M
SSD Mobilenet v2 lite	130	3.4M
SSD VGG16	50	35M
yoloV5 Nano	270	1.9M
yoloV5 Small	225	7.2M
yoloV5 Medium	109	21.2M
yoloV5 Large	72	46.5M
yoloV5 Extra Large	44	86.7M

**Table 7:** Average precision on test subset.

		Per Class mAP@0.5				
Model	Overall mAP@0.5	Conveyor Type 1	Conveyor Type 2	Rosy	Charging Station	Cone
SSD Mobilenet v1	0.860	0.909	0.891	0.816	0.717	0.969
SSD Mobilenet v2 lite	0.989	0.998	0.980	0.974	0.998	0.992
SSD VGG16	0.997	0.998	0.996	0.990	0.998	0.998
YoloV5 Nano	0.989	0.993	0.985	0.992	0.982	0.995
YoloV5 Small	0.992	0.995	0.989	0.994	0.99	0.995
YoloV5 Medium	0.994	0.995	0.992	0.995	0.991	0.995

## Data Availability

The obtained data set for object detection is publicly available.

## Abbreviations

The following abbreviations are used in this manuscript:

AP	Average Precision
APF	Artificial Potential Field
AR	Average Recall
CNN	Convolutional Neural Network
CPU	Central Processing Unit
FN	False Negative
FoV	Field of View
FP	False Positive
FPS	Frames Per Second
GPU	Graphics Processing Unit
GT	Ground Truth
IMU	Inertial Measurement Unit
IoU	Intersection over Union
IR	Infrared
LiDAR	Light Detection and Ranging
mAP	mean Average Precision
mAR	mean Average Recall
MEX	Matlab Executable
MS COCO	Microsoft Common Objects in COntext
OMR	Omnidirectional Mobile Robots
OROD	Omnidirectional Robot Object Detection
SP	Set Point
SSD	Single Shot Detector
TN	True Negative
TP	True Positive
TPU	Tensor Processing Unit
YOLO	You Only Look Once the algorithm
